# Geographic Access to Low‐Dose CT Lung Cancer Screening in Tennessee: Disparities by Socioeconomic Variables, Rurality, and Appalachian Status

**DOI:** 10.1111/jrh.70202

**Published:** 2026-08-08

**Authors:** Sima Namin, Martin Whiteside, R. Eric Heidel, Jonathan S. Wall, Jennifer Ferris, Rajiv Dhand

**Affiliations:** ^1^ College of Medicine—Knoxville Office of Research Support University of Tennessee Health Science Center Knoxville Tennessee USA; ^2^ Tennessee Department of Health Office of Cancer Surveillance Nashville Tennessee USA; ^3^ College of Medicine–Knoxville Department of Surgery University of Tennessee Health Science Center Knoxville Tennessee USA; ^4^ College of Medicine–Knoxville Department of Medicine University of Tennessee Health Science Center Knoxville Tennessee USA

**Keywords:** Appalachia, low‐dose computed tomography (LDCT), lung cancer screening, rural health, spatial access, Tennessee

## Abstract

**Introduction:**

Lung cancer screening enables earlier detection and improved outcomes, yet geographic inequities persist in access to low‐dose computed tomography (LDCT). We quantified tract‐level access to LDCT screening in Tennessee and examined disparities by rurality and Appalachian status.

**Methods:**

We compiled an inventory of LDCT‐capable sites and measured access using a 30‐min enhanced two‐step floating catchment area (E2SFCA) index with three banded decay weights. Access was compared across four strata (urban Appalachia, rural Appalachia, urban non‐Appalachia, and rural non‐Appalachia) using Kruskal–Wallis and pairwise Wilcoxon tests (Holm adjustment). Rank‐based associations between E2SFCA and tract sociodemographics were evaluated using Spearman correlations.

**Results:**

We identified 198 LDCT sites in Tennessee. Urban residents account for 74.10% of those living within ≤20 min, whereas rural residents constitute the majority (63.50%) of the 30–45‐min band. Access differed across strata (Kruskal–Wallis *χ* ^2^ = 277.46, *p *< 2.2 × 10^−16^): Medians (per 1000) were highest in urban non‐Appalachia (0.464) and urban Appalachia (0.430), and lower in rural Appalachia (0.210) and rural non‐Appalachia (0.197); all urban–rural contrasts were significant. Regional patterns diverged: West Tennessee showed the largest coverage gap (13.9% zero‐access tracts), East Tennessee had the lowest capacity among served tracts (median = 0.339; overall median = 0.293), and Middle Tennessee showed the strongest overall access (overall median = 0.380; 73/647 zero‐access; 11.3%).

**Conclusions:**

Rural communities face longer travel times to access LDCT screening sites with more zero‐access tracts than urban communities. Our findings support interventions such as adding service points in West Tennessee and expanding capacity in East Tennessee. Strengthening navigation and referral pathways for rural and Appalachian residents is also necessary.

## 1 Introduction

In the United States, lung cancer ranks second in incidence for both sexes (after prostate in men and breast in women), and it carries the highest mortality, accounting for nearly 20% of all cancer deaths in both sexes [1, 2]. In 2025, it is estimated that 226,650 new cases of lung cancer will be diagnosed in the United States, and 124,730 individuals will succumb to the disease [1]. Despite recent gains, the overall 5‐year relative survival is only 28.1% [3]. Mortality is falling nationally (4.2% per year since 2014), yet the disease's lethality keeps the population burden high [3].

Tennessee bears a disproportionately high burden of lung cancer. The incidence rate is 68.1 per 100,000 (vs. 53.1 the US average, 2017–2021), and the mortality rate is 44.1 per 100,000 (vs. 32.4 the US average, 2018–2022) [4]. These gaps reflect, in part, higher tobacco use (e.g., 17% of Tennessee adults were current smokers in 2023 vs. 12.1% nationally) [4]. Moreover, Appalachian counties not only experience substantially higher cancer incidence and death rates than the rest of the country [5], but they also experience longer delays from diagnosis to treatment [6].

Low‐dose computed tomography (LDCT) screening has been conclusively shown to reduce lung cancer mortality in high‐risk populations. In the National Lung Screening Trial (NLST), three annual LDCT screens led to a 20% reduction in lung cancer deaths compared with chest radiography [7, 8]. The Dutch Belgian NELSON trial further demonstrated a 24% relative reduction in lung cancer mortality at 10 years of follow‐up, with an even greater benefit observed among women (33% reduction) [9, 10]. Early detection via LDCT shifts diagnoses toward earlier stages, substantially improving 5‐year survival rates (from <20% for late‐stage disease to >60% for Stage I) and reducing the need for extensive treatment [11, 12].

Disparities in lung cancer early detection are stark across the United States. Uptake of LDCT screening remains under 20% among eligible, high‐risk Americans [9]. Geographic inequities exacerbate this gap: Individuals in rural areas face a 20% higher lung cancer incidence yet are far less likely to live near accredited screening centers. Approximately 90% of truly isolated rural residents lack a facility within 10 mi, compared with just 12% in urban settings [13, 14]. According to 2022 Behavioral Risk Factor Surveillance System (BRFSS) data, 19% of Tennesseans aged 50–79 years met the US Preventive Services Task Force (USPSTF) eligibility criteria for LDCT screening [15]. However, only 15% of those eligible underwent screening; under the American Cancer Society (ACS) guidelines, 26% were eligible, but only 14% were screened [15].

Access to LDCT is shaped by socioeconomic factors, geography, rurality, and insurance policy among others, producing clear disparities. Previous studies show that geography matters. For example, a national facility‐proximity analysis found that 97.6% of metropolitan residents had access to an LDCT site versus 41.0% of nonmetropolitan residents; longer travel times are also linked to lower completion of an initial LDCT even within urban safety‐net systems [16]. Studies have consistently shown lower screening uptake in rural areas and regional pockets of low access [17, 18]. Black and Hispanic adults remain less likely to be screened or diagnosed early [19].

Together, these data underscore the urgency of improving equitable access to LDCT screening, especially across rural and Appalachian communities in Tennessee. However, geographic proximity to an LDCT‐capable site represents only one component of lung cancer screening access. Evidence‐based lung cancer screening requires a coordinated program that includes identification of eligible patients, shared decision‐making, appropriate LDCT protocols, structured reporting, lung nodule management, smoking cessation support, patient and provider education, and program‐level data tracking [20, 21]. Prior work in rural settings has shown that facilities may offer LDCT scans without implementing all components of a comprehensive lung cancer screening program [22]. Therefore, spatial analyses of LDCT site availability should be interpreted as identifying geographic opportunities and constraints within a broader implementation pathway, rather than as direct measures of guideline‐concordant screening delivery.

This study provides a tract‐level assessment of geographic accessibility to LDCT‐capable sites across Tennessee. Specifically, we (1) compiled and validated a comprehensive inventory of LDCT‐capable screening sites from multiple sources, (2) quantified access using two complementary measures: travel time within 20/30/45/60 min and an enhanced two‐step floating catchment area (E2SFCA) index with step‐decay weights to capture both proximity and effective access; (3) tested disparities across rurality and Appalachia; and (4) examined Spearman's correlations with sociodemographic variables including education, racial/ethnic composition, and poverty. Our results will inform the placement of fixed and mobile screening resources where disparities and travel barriers are greatest.

## 2 Methods

### 2.1 Data Sources

#### 2.1.1 Low‐Dose CT Screening Facility Inventory

We assembled a master list of Tennessee LDCT‐capable sites from three sources: (1) The American College of Radiology (ACR) Lung Cancer Screening Registry (LCSR) program directory [23], (2) Medicare provider data identifying billers of LDCT screening [24], and (3) the Tennessee Health Facilities Commission's “*Health Care Providers that Utilize Computed Tomography Scanners‐Provider Addresses* (10/2/2024) file” and its associated CT equipment listings [25]. Because mobile capacity can materially alter access in rural/Appalachian areas, we also collected data on mobile CT programs operating in Tennessee via health‐system announcements and web searches.

#### 2.1.2 Population and Eligibility (Demand)

Tract‐level denominators came from the American Community Survey 2019–2023 5‐year estimates [26]. We constructed counts for adults ages 50–79 years from detailed age tables.

#### 2.1.3 Smoking Prevalence

To construct the demand measure, we used the Centers for Disease Control and Prevention (CDC) PLACES tract‐level estimates [27]. These estimates are modeled from the 2022 BRFSS survey responses and provide small‐area estimates for health indicators, including “current cigarette smoking among adults.”

#### 2.1.4 Geography and Classifications

The unit of analysis was the 2020 Census tract. Tract boundaries and identifiers were obtained from the Topologically Integrated Geographic Encoding and Referencing (TIGER)/Line 2020 files. Rural classification was obtained from RUCA (Rural–Urban Commuting Area) codes [28]. Appalachian status was added using the Appalachian Regional Commission (ARC) county list [29].

### 2.2 Analysis

To build the LDCT facility inventory, we used the ACR LCSR as the primary source. Because participation in the registry has not been required for Centers for Medicare & Medicaid Services coverage since February 2022, the registry does not capture all LDCT service locations. We, therefore, supplemented the registry using two additional sources: (1) 2023 Medicare provider data to identify locations that billed for LDCT screening using CPT/HCPCS code 71271 and (2) the Tennessee Health Facilities Commission's provider‐address and CT equipment files to identify CT‐capable facilities statewide. LDCT availability among facilities identified through the state files was confirmed through website review or telephone contact. We then combined and de‐duplicated the records and removed entries that represented provider billing addresses rather than physical LDCT service locations. To reduce edge effects in spatial analysis, we also incorporated facilities in neighboring states by using ACR and Medicare provider data to include sites within a 60‐min drive of the Tennessee border. The final list was geocoded in R using tidygeocoder package [30], which queries Esri's ArcGIS World Geocoding Service.

Tennessee census‐tract centroids were flagged for rurality and Appalachian status. Primary RUCA codes 1–3 were classified as urban, and 4–10 were classified as rural. Tracts were flagged as “Appalachian” if they fell within an Appalachian county using the ARC 2021 list.

Drive times and isochrones were computed on the OpenStreetMap (OSM) road network using OpenRouteService tool [31] in QGIS [32]. We generated 20, 30, 45, and 60‐min isochrones around all LDCT facilities and flagged tract centroids by their highest access band. These classifications supported a descriptive analysis of statewide LDCT access stratified by urbanicity and Appalachian status.

### 2.3 E2SFCA Index

Our primary outcome is the E2SFCA index across tracts. E2SFCA is widely applied in health services research because it accounts for both competition for supply and diminishing accessibility with travel time, providing a more realistic measure of service availability than simple proximity metrics [33]. E2SFCA method extends the classic 2SFCA accessibility framework by weighting opportunities within a catchment to reflect distance (or travel‐time) decay, addressing the unrealistic assumption of uniform access inside a threshold.

To create the E2SFCA index, we modeled LDCT screening accessibility for Tennessee census tracts, allowing cross‐border access to out‐of‐state facilities within 60 min of the Tennessee border (projected catchments subsequently constrained to 30 min). In Step 1, each facility's supply is divided by a weighted sum of nearby population demand. In Step 2, each population location accumulates the (weighted) ratios from all reachable facilities.

#### 2.3.1 Estimation of Tract‐Level Demand

For each tract *i*, *Di* demand was defined as the 2019–2023 ACS 5‐year population ages 50–79 years (female + male), the primary eligible LDCT screening ages, multiplied by the tract‐level prevalence of “Current cigarette smoking among adults” from CDC PLACES.

#### 2.3.2 Estimation of Tract‐Level Supply

Each LDCT facility *j* was treated as one unit of supply (S*j* =  1) because verifiable, site‐specific screening capacity was unavailable.

For network travel times and catchments, we computed road travel times (minutes) between all tract origins and facility destinations using the Open‐Source Routing Machine (OSRM) with OSM data. We accessed OSRM via the R osrm package [34], which exposes the OSRM table API for origin‐destination matrices. Origin‐destination pairs exceeding 30 min were excluded from E2SFCA.

#### 2.3.3 Decay Specification and E2SFCA Computation

The 0–30 min catchment was partitioned into three bands: 0–10, 10–20, and 20–30 min with nonincreasing weights *w*  =  {1.00, 0.68, and 0.22} applied to demand in Step 1 and to facility ratios in Step 2. These banded weights approximate distance‐decay without requiring parametric calibration and are widely used in E2SFCA studies [34].

Step 1 (facility ratio): For each facility, $Rj=\frac{Sj}{\sum_{i\in Cj}Di\;wij}$ where *Cj* is the 30‐min catchment of *j*, and *wij* is the band‐specific weight for travel time from *i* to *j*.

Step 2 (tract accessibility): For each tract *i*, $Ai=\sum_{j\in Ci}Rj\;wij$ where *Ci* is the set of facilities within 30 min of *i*. The scalar *Ai* is interpretable as supply per unit demand accessible to tract *i* under the specified decay. All processing was conducted in *R* using sf [35] for vector handling, osrm for travel‐time matrices, and ggplot2 [36] and classInt [37]. We classified the results of the access index into quintiles, with tracts lacking any sub‐30‐min access displayed as “No access (≥30 min).”

### 2.4 Statistical Analysis

We compared tract‐level LDCT accessibility across four groups (urban Appalachia, rural Appalachia, urban non‐Appalachia, and rural non‐Appalachia) using a nonparametric framework. Overall differences were tested with the Kruskal–Wallis test. Pairwise contrasts were evaluated with Wilcoxon rank‐sum (Mann–Whitney) tests, controlling the familywise error rate via the Holm procedure. We report Holm‐adjusted *p* values alongside group medians and confidence intervals for the accessibility index. Where relevant, we provide Hodges–Lehmann median shifts with 95% CIs as effect sizes.

Next, we summarized tract‐level accessibility to LDCT screening facilities using the E2SFCA method with a 30‐min driving catchment and distance‐decay weighting. Socioeconomic and demographic covariates at the census‐tract level included the percentages of residents with a high school education or higher, with bachelor's degree or higher, and living below the federal poverty level, as well as racial and ethnic composition, measured as the percentages of residents who were non‐Hispanic White, non‐Hispanic Black, Hispanic, or another non‐Hispanic race or ethnicity. Spearman rank correlations (*ρ*) were then estimated between access index and each variable. We reported correlations (1) overall, (2) stratified by urbanicity (urban vs. rural), and (3) stratified by Appalachian status (Appalachia vs. non‐Appalachia).

## 3 Results

**FIGURE 1:**
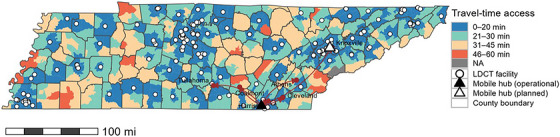
LDCT access bands and the distribution of fixed and mobile facilities. LDCT, low‐dose computed tomography.

One hundred and ninety‐eight (*n*  =  198) facilities with LDCT service were geocoded across Tennessee along with an additional 59 facilities in the neighboring states that were within 60‐min drive to Tennessee. The eligible population for LDCT in Tennessee was 442,140. Of these, 71.11% resided ≤20 min from an LDCT facility, 18.35% lived 21–30 min, 9.35% lived 31–45 min, and 1.18% lived 46–60 min away. Thus, about 9 in 10 eligible Tennesseans were within 30 min, 99% within 45 min, and effectively all within 60 min of an LDCT facility. Urban residents accounted for 74.10% of those living within ≤20 min, whereas rural residents constituted the majority of those living beyond 30 min (e.g., 63.50% of the 30–45‐min band) of an LDCT facility. In short, whereas near‐term access is high statewide, longer travel times were disproportionately high for rural communities. As shown in Figure 1, two mobile LDCT units are mapped statewide, one operational and based in Chattanooga (serving Athens, Harrison, Coalmont, Cleveland, Knoxville, and Tullahoma) and one based at UT Medical Center‐Knoxville that is planned to launch in Fall 2025.

Tract‐level LDCT accessibility differed significantly across the four geographic strata (Kruskal–Wallis *χ* ^2^  =  277.46, *p* < 2.2 × 10^−16^). Median accessibility was highest in urban non‐Appalachia (median  =  0.464; 95% CI, 0.442–0.480) and urban Appalachia (median  =  0.430; 95% CI, 0.393–0.461), and substantially lower in rural Appalachia (median  =  0.210; 95% CI, 0.163–0.227) and rural non‐Appalachia (median  =  0.197; 95% CI, 0.160–0.242).

Pairwise Wilcoxon tests with Holm adjustment confirmed urban–rural gaps (all urban vs. rural *p *< 0.001). Within rural tracts, Appalachia versus non‐Appalachia did not differ (*p*  =  0.997). Within urban tracts, access was higher in urban non‐Appalachia versus urban Appalachia (Hodges–Lehmann shift  =  −0.055; 95% CI, −0.092 to −0.019; *p*  =  0.00327).

**FIGURE 2:**
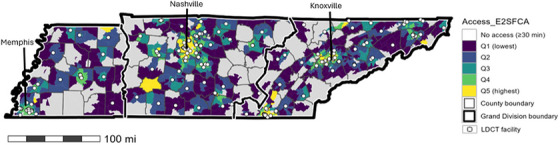
LDCT accessibility across Tennessee (E2SFCA, 30‐min, three band decay). E2SFCA, enhanced two‐step floating catchment area; LDCT, low‐dose computed tomography.

Figure 2 showed the choropleth map of tract‐level accessibility using E2SFCA with a 30‐min catchment and three‐band distance decay. Tracts were divided into quintiles of access, with a separate “No access (≥30 min)” class shown in gray. Accessibility was highly clustered around major metro areas: Nashville, Knoxville, Chattanooga, and Memphis, where many neighboring tracts fall in the top two quintiles. Access was not uniform across Tennessee's regions. Statewide, 1707 tracts were evaluated, and *n*  =  202 (11.8%) had no LDCT access within 30 min. Middle Tennessee had the strongest overall access. Although 73/647 tracts lack access (11.3%), it had the highest access intensity among served tracts (median  =  0.424) and the highest overall median when zero‐access tracts were counted as zero (median  =  0.380). West Tennessee's main gap was coverage; it had the largest share of zero‐access tracts (61/440; 13.9%); among served tracts, the median was 0.374, and the overall median was 0.332. East Tennessee's primary challenge was lower capacity relative to estimated demand. Its proportion of zero‐access was similar to that of Middle Tennessee (68/620; 11.0%), but its access intensity among served tracts was the lowest (median  =  0.339 per 1000), yielding the lowest overall median (median  =  0.293). It should be mentioned that estimated eligible population for LDCT (total  =  442,140) is not distributed evenly across the state with East Tennessee hosting 179,314 (40.6%), Middle 162,554 (36.8%) and West 100,272 (22.7%).

Across Tennessee census tracts, LDCT accessibility (E2SFCA) showed clear, rank‐based associations with tract social‐demographic composition (Table 1). These nonparametric correlations are intended for descriptive purposes; they do not imply causation. Education was positively related to access: The percentage of adults with a bachelor's degree or higher had a moderate correlation overall (*ρ*  =  0.404, *p* < 0.001), with similar positive associations in urban and rural tracts and a stronger pattern in Appalachia (*ρ*  =  0.455, *p* < 0.001) than non‐Appalachia (*ρ*  =  0.361, *p* < 0.001). The percentage of adults with a high school education or higher showed a smaller but significant positive correlations overall (*ρ*  =  0.176, *p* < 0.001). Tracts with higher percentages of non‐Hispanic Black residents had positive correlations with access. This association was markedly stronger in Appalachia (*ρ*  =  0.547, *p* < 0.001) than in non‐Appalachia (*ρ*  =  0.224, *p* < 0.001). In contrast, the non‐Hispanic White share was negatively correlated with access (overall *ρ*  =  ‐0.454, *p* < 0.001), a pattern present in both urban and rural areas and stronger in Appalachia than in non‐Appalachia. Poverty showed a negligible overall correlation with access (*ρ*  =  0.017, *p*  =  0.42); within urban tracts there was a small positive correlation (*ρ*  =  0.106, *p* < 0.001), whereas rural, Appalachian, and non‐Appalachian strata showed no meaningful association.

**TABLE 1:** Spearman correlations (*ρ*) between low‐dose computed tomography (LDCT) access (E2SFCA) and tract‐level characteristics.

Characteristic	Overall (*n* = 1505)	Urban (*n* = 1140)	Rural (*n* = 365)	Appalachia (*n* = 635)	Non‐Appalachia (*n* = 870)
% Bachelor's or higher	0.404***	0.323***	0.219***	0.455***	0.361***
% HS or higher	0.176***	0.071*	0.145**	0.249***	0.106**
% below poverty	0.017	0.106***	0.040	0.029	0.020
% Black (NH)	0.393***	0.286***	0.187***	0.547***	0.224***
% Hispanic	0.295***	0.252***	0.250***	0.319***	0.259***
% Others (NH)	0.288***	0.265***	0.146**	0.358***	0.219***
% White (NH)	−0.454***	−0.336***	−0.278***	−0.586***	−0.311***

*Note*: Spearman correlations (*ρ*) with LDCT access (*p < 0.05, **p < 0.01, ***p < 0.001).

Abbreviations: E2SFCA, enhanced two‐step floating catchment area; LDCT, low‐dose computed tomography.

## 4 Sensitivity Analysis

To assess the influence of facility capacity, we repeated the E2SFCA analysis assuming hospitals had double the screening capacity of imaging and diagnostic centers (weights: hospital  =  2, imaging/diagnostic/positron emission tomography (PET)/CT  =  1). The resulting spatial pattern was highly concordant with the uniform‐capacity model (Figure S1). “No‐access” tracts and regional rank ordering remained unchanged. Capacity weighting modestly amplified urban–rural contrasts: metro areas with higher hospital density showed broader high‐access zones (Q4–Q5), whereas rural tracts served only by imaging centers shifted downward (from Q3–Q4 down to Q1–Q2).

Correlations between access and sociodemographic characteristics were directionally consistent but attenuated (Table S1). For example, the correlation between education and access decreased from *ρ*  =  0.404 to *ρ*  =  0.336 overall, whereas a small positive association between poverty and access emerged in urban tracts (*ρ*  =  0.146, *p* < 0.001), reflecting clustering of high‐capacity hospitals in urban centers. Median accessibility increased statewide, and regional gaps narrowed slightly, but the overall findings held: West Tennessee remained the region with the largest zero‐access footprint, and East Tennessee continued to exhibit the lowest access intensity among served tracts.

## 5 Discussion

Overall, the results indicate that urban tracts had markedly higher accessibility to LDCT facilities than rural tracts, whereas Appalachia was not a differentiating factor in rural settings but was associated with slightly lower accessibility in urban contexts. The geography of access is not uniform across Tennessee's Grand Divisions, and the types of constraints differ by region. Middle Tennessee exhibits the strongest overall access: Even though 11.3% of tracts have no sub‐30‐min access, the overall median (counting zero‐access tracts as zero) remains highest statewide. West Tennessee's principal deficit is coverage: It has the largest share of zero‐access tracts (13.9%). East Tennessee's challenge is more capacity‐related with the highest percentage of eligible population compared to other regions: the zero‐access share (11.0%) is comparable to Middle Tennessee, but the access intensity among served tracts is the lowest, resulting in the lowest overall median.

Poverty showed minimal or no correlation after false discovery rate (FDR) control, particularly outside urban areas. The pattern persisted within Appalachia and non‐Appalachia and within each urbanicity stratum, with the strongest gradients observed in urban Appalachia. Overall, access tended to be higher in tracts with greater educational attainment and higher proportions of non‐White residents, and lower in tracts with larger non‐Hispanic White populations, with several relationships amplified within Appalachia. Sensitivity analyses incorporating capacity weights yielded consistent spatial patterns (Table S1, Figure S1), supporting the robustness of regional disparities identified under the uniform‐capacity model.

It should be mentioned that although tract‐based accessibility was higher in tracts with greater proportions of non‐White residents, this pattern likely reflects urban concentration of both minority populations and healthcare infrastructure rather than true equity in screening participation. Thus, spatial proximity alone does not ensure access, and non‐geographic barriers, such as awareness, provider referral, insurance coverage, and trust in the medical system, likely sustain further disparities in screening utilization.

From a planning standpoint, the results support a regional approach to address disparities in access to LDCT. West Tennessee would benefit most from new points of service (fixed or mobile) to reduce the zero‐access footprint, especially in rural counties. East Tennessee requires added capacity and scheduling where services exist, for example, extending scanning hours, adding mobile days, or increasing slots at existing sites, to lift access intensity for currently “served” tracts. Middle Tennessee's configuration is comparatively strong, but continued attention to rural areas is warranted to prevent widening gaps as demand grows.

These findings should be interpreted in the context of the broader distinction between LDCT availability and implementation of comprehensive lung cancer screening. Although approximately 90% of the estimated eligible population lived within 30 min of an LDCT‐capable site, proximity alone does not ensure that eligible patients are identified, referred, counseled, screened, followed longitudinally, or connected to smoking cessation services. This distinction is particularly important in rural areas, where LDCT may be available as an imaging service but not embedded within a fully coordinated screening program. Many rural facilities offering LDCT do not perform all recommended program components, especially lung nodule management, integrated smoking cessation, and patient/provider education [22]. Thus, the present study should be viewed as identifying the geographic infrastructure on which screening programs could be built or strengthened, rather than as evidence that complete screening access has been achieved.

Although this study is specific to Tennessee, its broader contribution is to show how spatial access metrics can be used to differentiate distinct rural screening–planning problems that may otherwise be obscured by simple distance‐to‐site measures. In Tennessee, most of the estimated eligible population lived within 30 min of an LDCT‐capable site, yet rural tracts still had lower accessibility, West Tennessee had a larger zero‐access footprint, and East Tennessee had lower access intensity among served tracts. These patterns suggest that the same statewide screening system may require different interventions in different regions: New fixed or mobile LDCT service points where geographic coverage is limited, expanded scheduling capacity where facilities exist but demand is high, and stronger referral, navigation, smoking cessation, and nodule follow‐up infrastructure where LDCT availability does not necessarily indicate a comprehensive screening program. This distinction is relevant to other rural and high‐burden states because planning based only on the presence or distance of LDCT sites may overestimate effective screening access.

## 6 Limitations

We integrated three data files to compile a list of facilities that offer LDCT, although we verified addresses to only keep service sites. Other misclassifications might occur due to temporary closures or changes in facilities’ provider policies for accepting patients. We treated each site as one unit of supply (*Sj*  =  1) due to unavailable, site‐specific capacity; true scanner count, and appointment availability vary. We could not account for payer authorization barriers.

We relied on OSM‐based drive times and a 30‐min catchment with three step‐decay bands (weights 1.00/0.68/0.22). Different thresholds, decay functions, or traffic assumptions would change absolute E2SFCA values, though relative patterns are typically robust. Eligibility was estimated with a proxy measure as age 50–79 years multiplied by CDC PLACES current‐smoking prevalence. This method does not capture former smokers who quit within the past 15 years, who are also eligible for LDCT under USPSTF/ACS guidelines. Because PLACES provides modeled small‐area estimates only for current smoking and not for pack‐years or time‐since‐quit, our demand measure likely underestimates the true screening‐eligible population and should be interpreted as a lower bound approximation.

Analyses are at the tract level and describe availability, not actual utilization or outcomes. The Spearman correlations are descriptive and unadjusted for spatial autocorrelation; although we stratified by urbanicity and Appalachian status and controlled FDR (Benjamini–Hochberg), spatial dependence may still inflate significance. Therefore, the results should not be interpreted as causal. Finally, three tracts were excluded from certain subgroup tables due to missing SES fields when all variables were required simultaneously.

Using census tracts rather than ZIP codes provides a methodological advantage for assigning rural–urban status. This design minimizes boundary mismatches that can occur when ZIP codes overlap multiple counties.

Importantly, our facility inventory captured LDCT‐capable service locations, not the presence, quality, or completeness of lung cancer screening programs. We could not determine whether each site provided shared decision‐making, standardized Lung‐RADS reporting, lung nodule tracking, smoking cessation counseling, patient navigation, annual rescreening reminders, or program data management. Therefore, our estimates should be interpreted as geographic accessibility to LDCT‐capable sites rather than access to comprehensive, guideline‐concordant lung cancer screening.

## 7 Conclusion

LDCT screening availability in Tennessee is, not surprisingly, spatially concentrated around major metros and uneven across regions. Approximately 90% of the eligible population lives within 30 min of a site, yet 11.8% of tracts have no sub‐30‐min access, and rural communities disproportionately face longer travel times. Education correlates positively with access, whereas the non‐Hispanic White share correlates negatively, patterns that likely reflect urban–rural placement of imaging resources rather than group‐specific barriers per se.

To translate access into timely utilization, Tennessee could pursue a dual strategy: (1) expand the number of service points (fixed or mobile) in West Tennessee to shrink zero‐access areas, and (2) strengthen capacity and scheduling in East Tennessee to raise access intensity where services already exist. Embedding mobile LDCT in rural hospital networks, coordinating cross‐border options, optimizing appointment windows, and sustaining patient navigation for shared decision‐making are practical levers to increase screening uptake, promote earlier detection, and reduce lung‐cancer mortality in high‐burden regions. Linking these spatial findings with stage‐at‐diagnosis and survival data will further identify where improved access can yield the greatest population‐level benefit.

Capacity weighting in the sensitivity analysis confirmed the robustness of the primary conclusions: Geographic coverage gaps persist in West Tennessee, whereas capacity‐related limitations dominate in East Tennessee, highlighting the need for both new rural service points and increased scheduling capacity in existing high‐burden areas.

## Funding

The authors have nothing to report.

## Ethics Statement

This project was exempt from approval by the University of Tennessee Health Science Center Review Board.

## Conflicts of Interest

The authors declare no conflicts of interest.

## Supporting information




**Supporting Information file 1**: jrh70202‐sup‐0001‐SupMat.docx

## Data Availability

The data that support the findings of this study are available from the corresponding author upon reasonable request.
