# Supplementary material for: Geographic Access to Low‐Dose CT Lung Cancer Screening in Tennessee: Disparities by Socioeconomic Variables, Rurality, and Appalachian Status
Source: J Rural Health. 2026 Aug 8;42(3):e70202. doi: 10.1111/jrh.70202 (PMC13452069; doi:10.1111/jrh.70202)
Supplement: Supplementary file 1 [file JRH-42-0-s001.docx]

**Supplements**

Figure S1: LDCT Accessibility across Tennessee

(weights: hospital = 2, imaging/diagnostic/PET/CT = 1)

Table S1: Spearman correlations (*ρ*) between LDCT access (E2SFCA) and tract-level characteristics

| Characteristic | Overall (n=1505) | Urban (n=1140) | Rural (n=365) | Appalachia (n=635) | Non-Appalachia (n=870) |
| --- | --- | --- | --- | --- | --- |
| **% Bachelor's or higher** | 0.336*** | 0.283*** | 0.196*** | 0.406*** | 0.286*** |
| **% HS or higher** | 0.110*** | 0.025 | 0.127* | 0.202*** | 0.037 |
| **% below poverty** | 0.075** | 0.146*** | 0.069 | 0.064 | 0.090** |
| **% Black (NH)** | 0.357*** | 0.287*** | 0.173** | 0.520*** | 0.247*** |
| **% Hispanic** | 0.261*** | 0.224*** | 0.233*** | 0.324*** | 0.206*** |
| **% Other (NH)** | 0.247*** | 0.226*** | 0.140* | 0.346*** | 0.166*** |
| **% White (NH)** | -0.418*** | -0.339*** | -0.264*** | -0.574*** | -0.316*** |

Spearman correlations (ρ) with LDCT access (* p<0.05, ** p<0.01, *** p<0.001)
